# Increased Gustatory Response Score in Obesity and Association Levels with IL-6 and Leptin

**DOI:** 10.1155/2016/7924052

**Published:** 2016-06-16

**Authors:** Nesrine Remla, Zeyneb Hadjidj, Kamel Ghezzaz, Soraya Moulessehoul, Mourad Aribi

**Affiliations:** ^1^Laboratory of Applied Molecular Biology and Immunology, University of Tlemcen, 13000 Tlemcen, Algeria; ^2^Stomatology and Oral Surgery Department of Tlemcen, University Medical Centre, 13000 Tlemcen, Algeria; ^3^Laboratory of Biotoxicology, University of Sidi Bel-Abbès, 22000 Sidi Bel-Abbès, Algeria

## Abstract

*Background*. The aim of this study was to investigate the relationship between the circulating IL-6 and leptin levels with taste alteration in young obese patients.* Methods*. A retrospective case-control study was conducted in thirty obese patients and thirty age- and sex-matched healthy controls.* Results*. Circulating levels of IL-6 and leptin were significantly increased in obese patients than in controls. However, catalase and ORAC levels were significantly decreased in obese patients compared to controls. Additionally, obese participants had high scores for the detection of fats (gustatory response scores [GRS]; *p* < 0.001). Moreover, IL-6 and leptin were strongly associated with GRS alteration among patients with GRS 4 (resp., OR =17.5 [95% CI, 1.56–193.32; *p* = 0.007]; OR = 16 [95% CI, 1.69–151.11; *p* = 0.006]). For the Mantel-Haenszel common odds ratio estimate (MH OR), IL-6 and leptin were strongly associated with obesity, in patients with either GRS 4 or GRS > 4 (resp., MH OR = 8.77 [95% CI, 2.06–37.44; *p* = 0.003]; MH OR = 5.76 [95% CI, 1.64–20.24; *p* = 0.006]).* Conclusions*. In a low grade inflammation linked to obesity, taste alteration is associated with high levels of IL-6 and leptin.

## 1. Introduction

The prevalence of obesity is reaching epidemic proportions and has become a global phenomenon, which is not only centred on the developed countries [1]. As per World Health Organization (WHO) estimates, the worldwide prevalence of obesity more than doubled between 1980 and 2014. In 2014, 39% of adults aged 18 years and over (38% of men and 40% of women) were overweight [2]. In the United States, more than 35.5% of men and 35.8% of women suffered from obesity in 2009-2010 [3].

North African countries and the region of Middle East just like the other developing countries are not spared from the issue of obesity. According to the Global Burden of Disease Study, these regions had the 7th highest prevalence of obesity in men (among the 21 GBD regions of the world) and the 2nd highest in women between 1980 and 2008 [1]. Algeria, Tunisia, and Morocco are part of the countries that are undergoing nutritional transitions to adapt themselves to Western lifestyles and to the demographic transitions and urbanisation. Recent data show that 14.9% of Moroccan, 29.6% of Tunisian, and 21.2% of Algerian populations suffer from obesity [4]. Another study in Algeria, conducted in 2010 by the Ministry of Health, Population and Hospital Reform, confirmed the extent of the disease in our society and stated the prevalence of obesity in both sexes at 21.24%. It is substantially higher in women than in men (30.08% versus 9.07%) [5].

Obesity is a multifactorial disease that combines both genetic and environmental factors [6], and it can be defined as an organ-associated pathology where adipose tissue plays a central role. The adipose tissue is constituted of several cell types that have the capacities of hypertrophy, hyperplasia, and differentiation [7]. It is able to secrete a number of endocrine and paracrine substances that have an important role in the immune system and inflammation, including interleukin-6 (IL-6) and leptin [8, 9].

IL-6 is a multifaceted pleiotropic cytokine, which may play a pivotal role in obesity. Since one-third of circulating IL-6 in healthy individuals is secreted by adipocytes, it has been termed adiponectin. Such cytokine regulates inflammation, haematopoiesis, immune responses, and hosts' defense mechanisms [10, 11]. The quantitative secretion of IL-6 from adipose tissue resulting in a systematic increase of its plasma levels may be apprehensive in proinflammatory status leading to a weight gain [12]. Both impaired IL-6 secretion and action are increased in obese individuals [13]. Besides, the correlation between the increased levels of IL-6 with adiposity and fat mass and not necessarily with insulin action has been shown in several studies [14]. Nevertheless, the implication of IL-6 in both pathologies is unresolved yet.

The adipose tissue has also been recognised as an active endocrine organ by secreting some molecules, especially leptin hormone among many other adipokines [10]. Leptin consists of 167 amino acid residue proteins, which have a broad range of effects on physiological processes and acting on the hypothalamic nuclei through its specific receptors [Ob-R]. It decreases appetite and increases energy expenditure through sympathetic activation, which consequently decreases adipose tissue mass and body weight [15, 16]. It has been reported that leptin stimulates dopamine uptake, creating a feeling of fullness. Furthermore, studies on leptin in both animal and human have shown that obesity is generally associated with elevated leptin levels [17], whereas leptin is said to play a dual role of inhibition/stimulation of lipogenesis and lipolysis, respectively, reducing intracellular lipid levels in skeletal muscle, liver, and pancreatic beta cells, thereby improving insulin sensitivity [18].

Several studies focused on the dietary habits show that obesity may result from an imbalance between food intake and food expenditure. Excess food consumption, particularly dietary fat, is taught to be one of the main causes of abnormalities linked to obesity [19]. It has been demonstrated that fats and other nutrients are detected by specific receptors in the mouth and gastrointestinal tract [20] and linoleic acid (C18:2) is one of the main fatty acids that are detected in the oral cavity [19].

Different protocols to determine the individual's oral fatty acid thresholds have been described in a number of published works in the last few years [21]; whatever the protocol and the composition of the solution used, the assessment of oral fatty acid detection is an important issue in obesity search.

The area of research concerning oral fatty detection is an emerging one in clinical investigation of obesity. We therefore tried to study for the first time the association that might exist between the circulating levels of IL-6 and leptin with the orosensitivity to linoleic acid. The current study was conducted in young Algerian subjects.

## 2. Materials and Methods

### 2.1. Patients and Subjects

Thirty obese patients (16 men; 14 women, age: 24.83 ± 2.8 years) and thirty healthy controls (15 men; 15 women, age: 24.56 ± 2.51 years) were recruited at the Laboratory of Applied Molecular Biology and Immunology of Tlemcen University. The main exclusion criteria were pregnancy, diabetes, hypertension, and cardiovascular disorders. The main inclusion criterion was the body mass index (BMI) ≥ 30. Both patients and subjects gave informed consent according to Helsinki's Declaration. This study was approved by the Institutional Ethics Committee of Sidi Bel-Abbes and Tlemcen.

### 2.2. Anthropometric Measurements

In order to avoid interpersonal variations while taking anthropometric measurements, only one person was in charge of this task during the whole study. Height and weight were measured by an automatic height-weight scale while patients were dressed in light clothing, without shoes. Waist and hip girth were measured according to the protocol described by Ross et al. [22], which have assessed the waist circumference at the point of the minimal waist while the hip circumference measurement should be taken at the widest portion of the buttocks.

### 2.3. Immunological and Biochemical Assays

#### 2.3.1. Blood Samples

Samples of peripheral venous blood were collected beginning at 8:00 a.m. after an overnight fast into BD* Vacutainer Venous Blood Collection* tubes containing ethylenediaminetetraacetic acid (EDTA, for plasma) or into tubes containing no anticoagulant (for serum). After centrifugation for 15 min, supernatants were transferred to new codified Eppendorf tubes, divided into aliquots, and stored at −80°C until use.

#### 2.3.2. Taste Sensitivity

The orogustatory perception of dietary lipids was evaluated through the use of the three-bottle preference test. Linoleic acid C18:2 was obtained from Sigma-Aldrich and was stored at −18°C. Fatty acids were added at varying concentrations, in an ascending order (0.018, 0.18, 0.37, 0.75, 1.5, 3, 6, and 12 mmol/L), in a solution of gum Arabica and demineralised water, to produce perceptually identical viscosity between fatty acid and control samples. To prevent oxidation of C18:2, all samples were mixed with 0.01% w/v EDTA. Samples were mixed by using sonication at 50% power with 30 seconds on then 60 seconds off for 9 minutes. An ice bath was used during sonication to control temperature. Samples were stored in opaque polypropylene cylinders and used within 48 hours of preparation. Control samples were prepared in the same way but without added fatty acids. On each trial, subjects were presented with three samples: two “blank” control samples and one containing the linoleic acid in ascending order of concentration from the lowest (0.02 mmol/L) to the highest (12 mmol/L). The subject's detection thresholds refer to the concentration of fatty acid required to correctly identify the “odd” sample from the two control samples and that in three consecutive sample sets. All participants wore nose-clips during the tests, in order to avoid confounding from nonoral sensory inputs (smells).

#### 2.3.3. IL-6 Assay

Plasma IL-6 was measured using human quantitative IL-6 enzyme-linked immunosorbent assay (ELISA) kit, as per the manufacturer's instructions (R&D, Sigma-Aldrich). The absorbance was measured at 450 nm using a microplate reader (Biochrom Anthos 2020, UK).

#### 2.3.4. Leptin Assay

The human plasma leptin was measured at 450 nm using appropriate ELISA kit (R&D, Sigma-Aldrich), according to manufacturer's instructions.

#### 2.3.5. Malondialdehyde Assay

Malondialdehyde (MDA) is one of the biomarkers for oxidative stress. The assay procedure included solvents and reagents such as thiobarbituric acid, trichloroacetic acid, and HCl. In brief, the process depends on combining the aqueous solution of serum and the TBA/TCA/HCl reagent (1V/2V). The mixture is well homogenised and then placed in a boiling water bath for 15 minutes. Samples are then centrifuged and the absorbance was read at 535 nm.

#### 2.3.6. Catalase Assay

Catalase activity was measured following the method of Aebi et al. [23]. 0.1 mL of plasma was added to 0.1 mL of hydrogen peroxide (H_2_O_2_) and 0.1 mL of saline water. After a 5-minute incubation period, 0.1 mL of TiOSO_4_ was added and the rate of H_2_O_2_ consumption was measured spectrophotometrically at 420 nm.

#### 2.3.7. Total Antioxidant Capacity Assay

The overall capacity of plasma to scavenge oxygen radicals (ORAC) was determined in plasma according to the KRL (Spiral/KIRIAL, Dijon, France) biological test, based on the haemolysis resulting from the attack of radicals [24].

### 2.4. Statistical Analyses

Mean difference between two groups was performed by two-tailed Student's* t*-test. The association analysis was evaluated by odds ratio (OR) and corresponding 95% confidence interval (95% CI), using the 90th percentile in the nonobese control group as cut-off levels. A pooled estimate of the common OR and its confidence interval was obtained by the Mantel-Haenszel method. Statistical analysis was performed using SPSS software (version 16.0). *p* values less than 0.05 were considered statistically significant.

## 3. Results

The demographic characteristics of the obese patients and nonobese subjects are shown in Table 1.

No differences between the two groups were found regarding age and sex (for both comparisons, *p* > 0.05). However, BMI levels were significantly higher in obese participants compared with the age-matched normal weight subjects as to waist and hip circumferences (for the two comparisons, *p* < 0.001).

**Figure 1:**
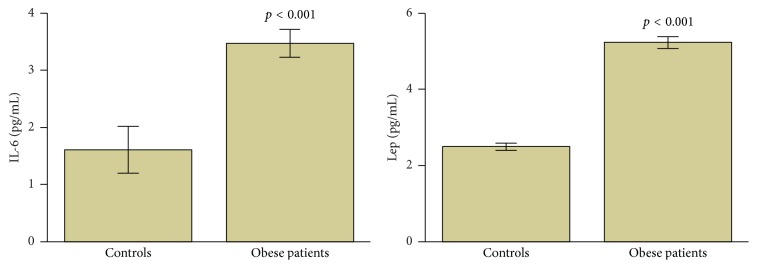
Circulating levels of IL-6 and leptin in obese patients and controls. *p* < 0.05 was considered statistically significant. Data are presented as mean ± standard error. IL: interleukin and Lep: leptin.

As shown in Figure 1, circulating levels of IL-6 and leptin were significantly increased in obese patients when compared to normal weight subjects (for the two comparisons, *p* < 0.001).

**Figure 2:**
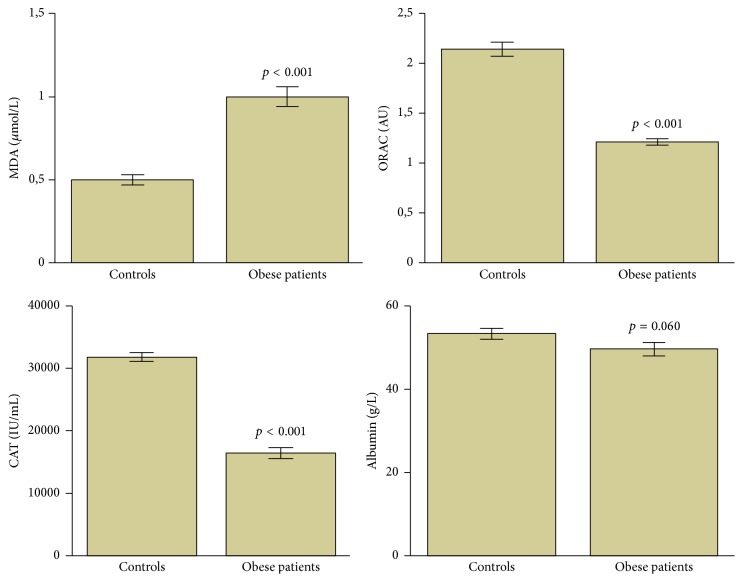
Oxidative stress biomarkers in obese patients and controls. *p* < 0.05 was considered statistically significant. Data are presented as mean ± standard error. MDA: malondialdehyde, ORAC: oxygen radical absorbance capacity/total antioxidant capacity, and CAT: catalase.

Additionally, catalase and ORAC levels were significantly decreased in obese patients compared to the age-matched controls (*p* < 0.001). Moreover, serum levels of albumin were decreased in obese patients compared to nonobese subjects, but the difference did not reach statistical significance level (*p* > 0.05). Conversely, MDA levels were significantly increased in obese patients than in nonobese subjects (*p* < 0.001) (Figure 2).

**Figure 3:**
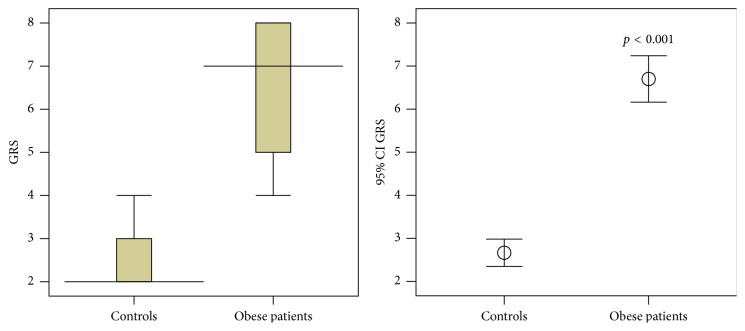
Results of gustatory response scores for the detection of fat in obese patients and controls. *p* < 0.05 was considered statistically significant. Data are presented as mean ± standard error. CI: confidence interval and GRS: gustatory response score.

Figure 3 shows that control subjects exhibited high oral sensitivity for linoleic acid (low detection threshold), while obese participants had high scores for the detection of fats (gustatory response scores [GRS]; *p* < 0.001).

**Figure 4:**
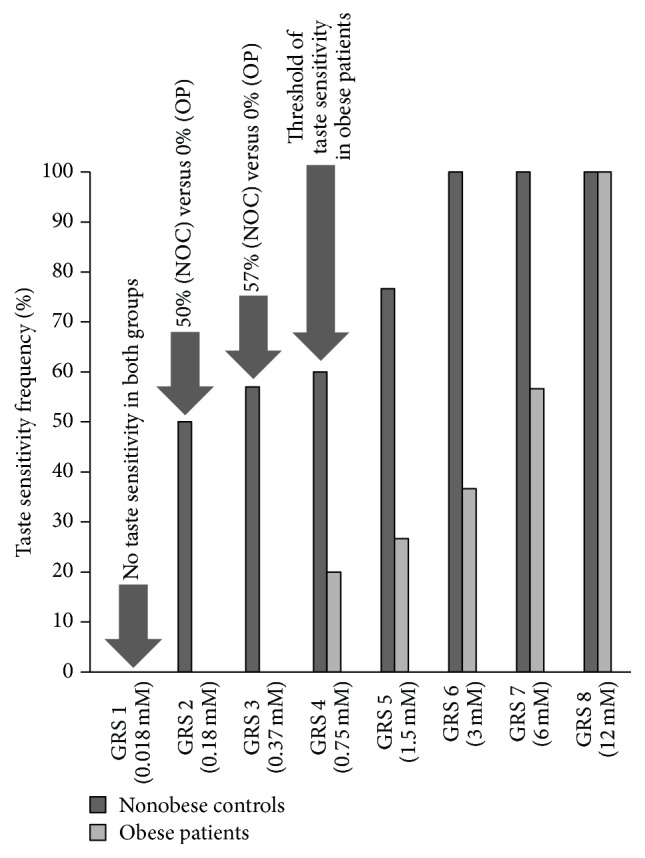
Threshold of taste sensitivity in obese patients and controls. Fat's detection thresholds in nonobese controls are perceptible starting from the second GRS, while obese patients could not taste fat before the fourth GRS. NOC: nonobese controls, OP: obese patients, and GRS: gustatory response score.

Additionally, the thresholds of GRS in control subjects and obese patients were 2 and 4, respectively (Figure 4).

We show in Figure 4 that both control and obese patients could not be able to detect the presence of fat in the lowest concentration, whereas almost all the volunteers detected the presence of fat before the third concentration. Few of the obese patients, however, detected fat starting from the fourth solution.

**Figure 5:**
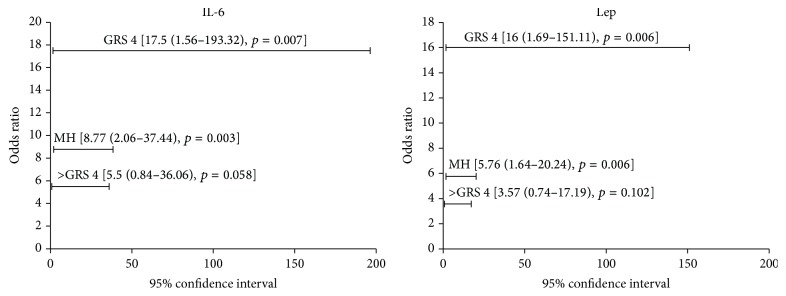
Association analysis of IL-6 and leptin with obesity among patients with and without taste alteration according to the GRS 4. Odds ratio and corresponding 95% confidence interval were calculated to determine association of IL-6 and leptin with obesity, using the 90th percentile in the nonobese control group as cut-off levels. The total number of patients with GRS 4 is 6. The numbers of patients with GRS 4 and increased circulating levels of IL-6 and leptin were 5 and 4, respectively. The total number of patients with GRS > 4 is 24. The numbers of patients with GRS > 4 and increased circulating levels of IL-6 and leptin were 22 and 20, respectively. IL: interleukin, Lep: leptin, MH: Mantel-Haenszel common odds ratio estimate, and GRS: gustatory response score.

We report in Figure 5 that IL-6 and leptin were strongly associated with obesity among patients with GRS 4 (resp., OR = 17.5 [95% CI, 1.56–193.32; *p* = 0.007]; OR = 16 [95% CI, 1.69–151.11; *p* = 0.006]). Nevertheless, such associations were not significant in patients with GRS > 4 (IL-6; OR = 5.5 [95% CI, 0.84–36.06; *p* = 0.058]; leptin; OR =3.57 [95% CI, 0.74–17.19; *p* = 0.102]). Finally, for the Mantel-Haenszel common OR estimate, IL-6 and leptin were strongly associated with obesity, in patients with either GRS 4 or GRS > 4 (resp., Mantel-Haenszel common OR estimate = 8.77 [95% CI, 2.06–37.44; *p* = 0.003]; Mantel-Haenszel common OR estimate = 5.76 [95% CI, 1.64–20.24; *p* = 0.006]).

## 4. Discussion

In epidemiological studies, one way to determine adult obesity in both sexes is BMI, which is determined as weight in kilograms divided by height in meters squared. Even though body mass index is the most common measure of obesity, it does not, however, allow appreciating the repartition of fat, which differs between men and women and even in the same sex among different populations [25]. For more accuracy in the definition of the body shape, that is, distribution of body fat, the measurement of waist circumference is necessary [26].

Fat may exist into two main compartments in the human body: visceral (torso) and subcutaneous (under the skin). In order to identify individuals morbidity related to obesity, World Health Organization (WHO, 2000) suggested the determination of other indicators of body fat distribution, mainly the waist-hip ratio (WHR). The ratio provides an index of both subcutaneous and intra-abdominal adipose tissue [27]. Hence, our study population was strongly different between obese and age-matched controls in terms of BMI and WHR.

Obesity is associated with an inflammatory-like status [28] where some cytokines are secreted either from the adipose tissue or from macrophages in the adipose tissue stroma [29] and which can be associated with numerous medical comorbidities. As mentioned above, adipose tissue is constituted of several cell types that are able of secreting a large number of physiologically active peptides that have common properties with cytokines [30]. Hypertrophic adipocytes secrete free fatty acids (FFAs) and contribute with the immune cells to the release of various proinflammatory cytokines, like IL-6 [31]. Cytokines can be released from a wide range of immune cells to act as mediators for a large number of immune responses. Some of them are directed to increase the immune system activity, while the others downregulate the immune responses, creating in this regard a balance between proinflammatory and anti-inflammatory mediators ensuring thereby homeostasis.

IL-6 is a proinflammatory cytokine with a molecular mass varying from 21 kDa to 28 kDa (184 amino acids), secreted by a number of different cells including activated macrophages, lymphocytes, fibroblasts, and endothelial cells, but also adipocytes. In humans, this protein is encoded by the IL6 gene, which mapped to 7p15–p21 chromosome and consists of five exons and four introns [32, 33]. This pleiotropic cytokine is involved in various physiological and pathophysiological processes, mainly inflammation, haematopoiesis, carcinogenesis, and the production of +ve acute phase proteins such as C-reactive protein (CRP) and C3 [34, 35]. Through diverse mechanisms, IL-6 plays important roles in the pathogenesis of inflammatory diseases and cancer [36]. The IL-6 signals start by binding a receptor composed of two different subunits, an alpha subunit that produces ligand specificity and glycoprotein (GP) 130, a receptor subunit common to all the members of the IL-6 family members. The binding of IL-6 to its specific receptor leads to a series of intracellular signalling cascade, involving the activation of Janus (JAK) tyrosine kinase family members and the activation of Ras-mediated signalling. Activated JAK kinases results in the activation and phosphorylation of signal transducer and activator of transcription (STAT) factors. Another major signalling pathway for IL-6-type cytokines is the mitogen-activated protein kinase (MAPK) cascade. Anomalies in IL-6-type cytokine signalling have been involved in the onset and maintenance of various inflammatory diseases [37].

In our study, circulating levels of IL-6 were increased in obese patients. It has been reported that increased IL-6 levels in metabolic dysregulations can promote adiposity [38]. In a study conducted on atherosclerosis, IL-6 has been shown to play a pathophysiological role altering lipoprotein lipase (LPL) activity and stimulating lipolysis [39].

Leptin is a 16 kDa pleiotropic protein [38, 39] mainly secreted by white adipose tissue [40] by the* ob* gene [41]. In addition to its metabolic and endocrine functions, leptin regulates energy expenditure and food intake through a direct effect on hypothalamus [41, 42]. It can also have a regulating action on haematopoiesis, innate and adaptive immune responses, and inflammation, especially with proinflammatory actions [41, 43]. Additionally, leptin and its receptor share structural and functional similarities with inflammatory cytokines, which suggests that leptin might be classified as a cytokine [40, 42].

Even though leptin is essential for normal immune response [44], its deficiency increases susceptibility to infectious and inflammatory stimuli and can lead to dysregulation in cytokines production [40]. Hyperleptinemia, referred to as a state of excess adiposity, is commonly seen in obesity and may play an important role in potentially serious health problems, such as cardiovascular diseases and rheumatoid arthritis [45].

As a modulator of immune and inflammatory responses, leptin induces cytokine transcriptional response in the cell [44]. Studies show that leptin leads to its action favoring the proliferation and production of proinflammatory cytokines by T cells, macrophages, and dendritic cells and the downregulation of proliferation and expansion of regulatory T (Treg) cells [46]. Leptin may also stimulate increasing acute phase proteins such as CRP [41]. Additionally, leptin acts through its specific receptor, Ob-R, which can exist in six isoforms in various cells, including immune cells, vascular smooth muscle cells, and endothelial cells. The longest isoform Ob-Rb of the leptin receptor, referred to as class I cytokine receptor, which is expressed by a large population of immune cells, is the only isoform that contains active intracellular signalling domains, that is, two cytokine-like binding motifs, Trp-Ser-Xaa-Trp-Ser (WSXWS), and a fibronectin type III domain [41, 44]. Three signal-transduction pathways can be elicited after binding leptin: JAK-STAT, PI3K, and ERK1/2 [43]. Moreover, it has been shown that Ob-Rb contains a glycoprotein (gp) 130 family of cytokines, which includes IL-6 [42].

It is now clear that oxidative stress is involved in the pathological processes of obesity. In the current study, we showed that obese patients have higher levels of MDA, which is one of the most important oxidative stress biomarkers. This can result in the development of several complications [47]. Thus, excess of nutrients and fatty acids cause dysregulation of carbohydrate and lipid metabolism by the lipid accumulation in nonadipose tissues with limited storage capacity [48, 49]. This lipotoxicity induces cellular stress and inflammation that lead to cell damage [48]. Overnutrition also leads to mitochondrial dysfunctions and generation of reactive oxygen species (ROS) [49] that lead to oxidative stress. It has been reported that oxidative stress can also be induced by adipocyte associated inflammatory macrophages [47]. Upon the increase of adipose tissue that secretes more proinflammatory adipocytokines that in turn generate more ROS, the activity of antioxidant enzymes was found to be significantly decreased in our obese patients as reported [50]. Originating locally at adipose depots, oxidative stress, endoplasmic reticulum stress, and inflammation are each involved in the progression of obesity-associated metabolic diseases [51, 52].

Despite the inflammatory condition of obesity, one hypothesis states that the onset of obesity may result from an imbalance between energy intake and energy expenditure. The alteration in the energetic homeostasis mainly caused by an excessive consumption of large amount of fat [53] leads some researchers to emphasize their studies on the oral chemosensory detection system for fatty acids in animal models and human. Mattes [54] highlighted the palatability of dietary fats to humans and the factors contributing to their attractiveness, in particular the olfactory and textural factors.

Gustatory responses scores obtained after an orosensory test to linoleic acid (C18:2) were useful to classify our participants into hypo- and hypersensitive to linoleic detection, as Stewart et al. [20] did with the oleic acid (C18:1). We show that hyposensitive subjects to linoleic acid had greater BMI compared to the hypersensitive. Our results corroborate others [53] regarding the low taste sensitivity. Accordingly, we postulate that oral sensitivity to dietary fats is compromised in obesity, which join the hypothesis that there may be a sixth taste modality in addition to sweet, sour, salty, bitter, and umami and which is only devoted to the perception of dietary lipids [55].

The present study investigated for the first time the oral sensitivity to linoleic acid (C18:2) in obese patients and its association with the circulating levels of IL-6 and leptin. There was a significant association between the alteration of the orosensory of linoleic acid and IL-6 and leptin. Additionally, hyposensitive patients with GRS 4 had significantly higher IL-6 and leptin levels.

## 5. Conclusions

This first report highlights that obesity combines an alteration in oral sensitivity to C18:2 with its association with an increased levels of IL-6 and leptin. The exact mechanisms associating these molecules in cluster during obesity remains largely elusive. We suggest that future studies demonstrate whether the compromised oral sensitivity to dietary fats is a cause or a consequence of the chronic state of inflammation in obesity. Additionally, it would be of a primary importance to isolate cells from the adipose tissue of obese with a taste disorder to assess any variation in their expression levels of both IL-6 and leptin. Moreover, the ideal would be to check whether such molecules were secreted by taste bud cells from the fungiform papillae in order to look for a possible cause-and-effect relationship with the fat taste receptors.

## Figures and Tables

**Table 1:** Characteristics of patients and subjects of the current study.

Variable	Nonobese controls *n* = 30	Obese patients *n* = 30	*p* value
Age (year)	24.57 ± 0.459	24.83 ± 0.512	0.700
Sex (M/F)	1.57 ± 0.092	1.47 ± 0.093	0.440
Weight (Kg)	57.72 ± 1.28	102.73 ± 2.5	<0.001
Height (m)	2.0 ± 0.0	2.03 ± 0.033	0.321
BMI (Kg/m^2^)	21.56 ± 0.33	35.42 ± 0.56	<0.001
Waist circumference (M and F, cm)	86.13 ± 1.71	110.1 ± 1.95	<0.001
Hip circumference (M and F, cm)	93.33 ± 1.41	115.99 ± 4.27	<0.001
WHR (M and F)	0.92 ± 0.01	3.15 ± 2.24	0.320

*p* < 0.05 was considered as significant. BMI: body mass index, M: male, F: female, WHR: waist-to-hip ratio.
